# Promising improvement of chronic lateral elbow tendinopathy by using adipose derived mesenchymal stromal cells: a pilot study

**DOI:** 10.1186/s40634-020-00320-z

**Published:** 2021-01-26

**Authors:** Miguel Khoury, Montassar Tabben, Alejandro U. Rolón, Lorena Levi, Karim Chamari, Pieter D’Hooghe

**Affiliations:** 1 Cleveland Orthopedics, Buenos Aires, Argentina; 2 Aspetar Qatar Orthopaedic and Sports Medicine Hospital, P.O. Box 29222, Doha, Qatar; 3 Himan MRI Institute, Buenos Aires, Argentina; 4 Regenerar Laboratory, Buenos Aires, Argentina

**Keywords:** Cell therapy, Tennis elbow, Tendon healing, Lateral epicondylitis, Racket sports

## Abstract

**Purpose:**

Study the effect of Adipose derived stromal cells (ASCs) injection as therapeutic procedure on the common extensor tendinopathy.

**Methods:**

Eighteen Tennis players with chronic, recalcitrant LET (who have previously been unsuccessfully treated with nonoperative treatments) underwent clinical evaluation and magnetic resonance imaging (MRI) before intervention. Stromal vascular fraction cells (SVF) were expanded by in vitro culture and ASCs were obtained and characterized by flow cytometry. ASCs were injected into the site of tendinopathy (identified by ultrasound imaging at the origin of the common extensor tendon) on a single occasion followed by physiotherapy. Players underwent serial clinical evaluations during a 12-month period and repeated MRI at 6-month post-injection.

**Results:**

At 6-month clinical evaluation revealed significant improvements compared to baseline in mean Visual Analog Scale (VAS) scores for: (1) maximum pain score (from 6.28 ± 1.65, to 1.0 ± 0.43; *p* < .001); (2) Mean quick Disabilities of the Arm, Shoulder and Hand (QuickDASH-Compulsory score: 51.38 ± 12.02 to 12.33 ± 4.66; *p* < .001); (3) QuickDASH-Sport score: 56.94 ± 15.44 to 8.68 ± 8.86; *p* < .001). Validated MRI scoring system grade of tendinopathy also improved significantly: 4.22 ± 0.26 to 2.22 ± 0.10 (*p* < .001). At 12-month from injection, VAS maximun pain score further decreased to 0.74 ± 0.44 (*p* < .001) and QuickDASH-Compulsory score to 5.56 ± 3.58 (*p* < .001). Average time to return to play tennis was 3,31 ± 0,61 month post-intervention.

**Conclusion:**

Tennis players with recalcitrant LET showed significant clinical improvement and structural repair at the origin of the common tendon origin after injection of autologous ASCs. Results of this study are promising and open a new biological therapeutic modality to treat LET. Even if the results of this pilot study are positive, future well-designed studies, i.e. prospective randomized trials are needed to define the role of cell therapy in treating LET.

## Background

Lateral elbow tendinopathy or tennis elbow (LET) is related to microtrauma that causes degenerative conditions in wrist extensor tendons. Its’ prevalence is 1%–3% in the general population [2]. Despite the condition being commonly referred to as tennis elbow, tennis players make up only 10% of the patient population. Half of mature amateur tennis players develop pain around the elbow, of which 75% represent true tennis elbow. Individuals that play racket sports involving repetitive wrist extension, radial deviation, and/or forearm supination are prone to this injury. LET is more common in players older than 40 years of age and main complaints are pain, decreased grip strength and impairment of functional activities, which may cause significant disability to play. Most of players with LET spontaneously recover within one year, even without intervention, the remaining are difficult to manage successfully, and unresolved symptoms lead to chronic disease [18].

It has been shown that microtrauma leads to depletion of tenocyte population due to apoptotic and autophagic cell death [6]. This further impairs collagen synthesis [21]. The ethiology of this disorder is related to a process resulting in vascular proliferation and hyaline degeneration of the extensor carpi radialis brevis and the extensor digitorum communis (common extensor tendon origin.). Most of individuals with LET spontaneously recover within one year, even without intervention, the remaining are difficult to manage successfully, and unresolved symptoms lead to chronic disease. Classic treatments include physiotherapy, and anti-inflammatory medication. Corticosteroid injections has been widely used, but most of the reports suggest that pain relief and effectiveness of the treatment is transient. Moreover, the use of corticosteroids decreases cellular activity of human tenocytes and collagen synthesis, increasing the long term weakening and risk of tendon’s rupture [9]. At present there is an increased understanding of the physiopathology of tendinosis and hence, new modalities of biological treatments are being used. Platelet Rich Plasma (PRP), autologous blood injections, extracorporeal shock wave therapy, dry needling, botulinum toxin, and prolotherapy were reported as being effective with different degrees of success [1, 15, 25]. In more than 90% of players LET is successfully treated nonoperative. For the minority of patients with refractory pain, surgery is an option [26].

Treatment of LET can be challenging once nonoperative measures fail. Breaking the cycle of tendon tearing and healing failure through biological stimulation is very attractive. Healing the tendon and minimizing scar tissue formation, the recovery of structural properties and reinforcement of tensile strength is the goal of biological treatments [38]. By definition, stromal/mesenchymal cells are able to self-renew and exist in every human tissues in an undifferentiated or unspecialized state, being able of differentiation or specialization along multiple lineages [3]. Adipose tissue is an excellent source of adipose derived mesenchymal stromal cells (ASCs). These have been receiving increasing attention over the years toward enhancing tendon healing [32]. Technically, ASCs can easily be isolated from subcutaneous adipose tissue, from liposuction aspirates or biopsy needle. Evidences from in vitro and in vivo studies suggest that ASCs can contribute to accelerate and improve the quality of tendons [36]. Co-culture studies demonstrated that the cellular crosstalk leads to an up-regulation of tendon-related genes, supporting a potential therapeutic effect for ASCs in tendinopathy [10]. Nevertheless, the exact mechanisms underlying these repair events are yet to be fully elucidated.

We hypothesized that ultrasound guided injections of autologous ASCs in chronic recalcitrant LET may be useful in healing the damaged tendon and further decreasing the need of surgery and a successful return to play. We also aimed to intrinsically evaluate adverse effects and complications of ASCs culture preparation as an alternative treatment of LET.

## Methods

This prospective longitudinal case series exploratory study was approved by the ethical committee of Regenerar^R^ Facility (Clinical Trial MR0012). Patients with LET who referred to our institution from January 2015 to February 2019 were invited to participate. All participants gave informed written consent. Before intervention, a detailed clinical history was performed and visual analog scale (VAS) for maximun pain, quick Disabilities of the Arm, Shoulder and Hand (QuickDASH) Compulsory, QuickDASH-Sport [16], X-rays and MRI to the involved elbow evaluations were performed. The diagnosis of LET was confirmed using MRI standard imaging protocol. The intervention consisted in a single dose of 7,9 × 10^6^ ASCs. The follow-up protocol included VAS for maximun pain, QuickDASH Compulsory and adverse events at 1, 3, 6 and 12 months. QuickDASH-Sport, and MRI were repeated only at 6 months. Patient satisfaction was evaluated at 3, 6 and 12 months using a scale including the following categories: very satisfied, satisfied, neutral, unsatisfied and very unsatisfied.

### Participants

A total of 19 patients (males = 11 and females = 8; age: 46.5 ± SD) provided consent to enter the study. Exclusion criteria were: lateral elbow pain from other musculoskeletal causes (arthritis, synovitis, posterior interosseous nerve entrapment, fibromyalgia), history of elbow instability or previous elbow surgery, any other pathology involving the affected upper limb or cervical spine pathology, full-thickness tear of the common extensor origin or any intra-articular pathology or chondral defects or Lateral collateral ligament tears al MRI and patient with contraindication to injection therapy, bleeding diathesis or on anti-coagulant, local or systemic infection, injections of corticosteroid medication in the previous 3 months. One female player withdrew consent 3 months after injection and had surgical treatment. All remaining 18 patients were followed during the 12 months’ period with no loss.

All participants had pain and disability for at least 4 months (Mean 7,38 SD3,2 months) and had been treated without success, by conventional treatments, including physical therapy using an eccentric rehabilitation protocol, and oral medication [18], Prolotherapy [2] three weekly injection protocol, extracorporeal shock wave therapy [3], three to five sessions using a standard LET protocol, steroid injection [10], unguided tendon injections with dipropionate and sodium phosphate of betamethasone. and platelet rich plasma [6], 3 weekly injections of leucocyte rich platelet rich plasma. No Patients have received mesenchymal cells due to any medical condition previously.

### Intervention

#### Collection of Adipose Tissue

Prior to collection, the patient had to have normal prothrombin time, partial thromboplastin time and platelet count. Adipose tissue was obtained from the periumbilical zone. The procedure was done in an outpatient setting using local anesthetic of 2% lidocaine with epinephrine. A total of 2–5 ml of adipose tissue was obtained in multiple punctures with (BARDR MAGNUMR) Reusable Core Biopsy Instrument and the corresponding Biopsy Needles. Samples were collected in sterile Hanks Balanced Salt Solution (HBSS) (Gibco, Cat # 14,025,088).

#### AT-MSC isolation and culture

Adipose Tissue (AT) were rinsed of Hanks Balanced Salt Solution (HBSS) (Gibco, Cat # 14,025,088) and incubated with collagenase (Collagenase from Clostridium histolyticum, SIGMA, Cat # C9722). MSCs were counted and seeded in T25 cm2 tissue culture flask in complete media supplemented with 100 IU penicillin, 100 IU anphotericin and 100 IU streptomycin (Ritcher), 10% autologous serum. When cultures reached 90–100% confluence, subculturing was performed using trypsin Tryple Select (Gibco, Cat. 12,563,029). At harvest, cells of all patients were in passages lower or equal to 3. Prior to injection, MSCs were tested for endotoxin, mycoplasma, and microbial contamination. For injection, MSCs were washed and suspended in 5 ml HBSS without antibiotics. Cell count and viability was confirmed using a manual hemocytometer. In the laboratory Regenerar^r^ facility ASCs were successfully grown in culture. Theese cells exhibit elongated nuclei and spindle-shaped cytoplasm, characteristic of a fibroblast-like morphology.

#### Characterization of ASCs

ASCs were stained with antibodies against CD73, CD90, CD105, CD45, CD34, CD11b, CD19 or CD79a, CD14, and HLA class II, to determine surfer marker expression. In accordance with the International Society for Cellular Therapy (ISCT) recommendations: ≥ 95% of the MSC population expressed CD105, CD73 and CD90, as measured by flow cytometry. Additionally, these cells lacked expression (≤ 2% positive) of CD45, CD34, CD14 or CD11b, CD79a or CD19 and HLA-DR (class II.20) Also, the cells were able to differentiate to osteoblasts, adipocytes and chondroblasts under standard in vitro differentiating conditions [12].

FACSCanto II BD flow cytometer instrument was used for running samples. The samples were immunostained with a determined panel of monoclonal antibodies (mentioned above) conjugated with different fluorochromes and mouse isotype antibodies were used as control. The results were analyzed with the software Infinicyt version 1.7i.

#### Percutaneous ultrasound guided intra-lesional delivery of ASCs

ASCs were injected at an average of 1 month after tissue collection. The cultured ASCs were washed twice and suspended in 3 ml of Hanks Balanced Salt Solution (HBSS) (Gibco, Cat # 14,025,088). Prior to the injection, the skin was prepared by aseptic technique with iodine solution. Approximately an average of 7,9 × 10^6^ ASCs were injected in each affected elbow. The cells were delivered percutaneously under local anesthesia. 2% lidocaine was previously superficially injected to numb the skin. An ultrasound equipment (Esaote X6, L 4–15 MHz probe) was used to guide injections into the hypoechogenic area and were performed by the same physician (MAK).

#### Postprocedure Protocol

Immediately after the injection, the patient was kept in a supine position without moving the arm for 15 min. Patients were sent home with instructions to rest the arm for approximately 24 h. If necessary, patients were allowed to use acetaminophen, but use of nonsteroidal anti-inflammatory medication was not allowed. After 48 h, patients were given a standardized stretching protocol to follow for 2 weeks under the supervision of a physiotherapist. A formal eccentric muscle- and tendon-strengthening program was initiated after this stretching program. At 1 month after the procedure, patients were clinically evaluated to determine the proceeding with normal sporting or recreational activities thereafter individually indicated.

### Magnetic resonance imaging

The equipment used was a 1.5 T MRI scanner (Philips, Eindhoven, The Netherlands) Flex-M Coil. STIR Coronal, Axial & Sagital view (FOV 130 mm, TR 3500, TE 50, TI 100) Thickness 3 mm. The radiological interpretation was performed by two experienced sports trauma radiologists. We used a standardize and reproducible scoring system. This score system grades the tear (score 1–3) and tendinopathy (score 1–3). The final score goes from 2 to 6. A final score of 2 indicates a tear < 25% of the tendon thickness and complete homogeneous low intensity or mild focal increased tendon signal on MRI, while a final score of 6 indicates severe tendinopathy with a tear > 50% of the tendon thickness and generalized increased signal intensity on MRI [39].

### Statistics

Data were processed with R software (R Foundation for Statistical Computing, Vienna, Austria. URL https://www.R-project.org/). Normality of the distribution of numerical variables was verified using the Wilk-Shapiro test. Values are informed as mean ± standard deviation (SD). Given that data was available for all variables at that time, the results at 6 months were compared to the pre-intervention values using Paired t test and Mean differences with 95% Confidence Interval (CI) of the mean difference.

To evaluate the effect of changes over the time at 1, 3, 6 and 12 months in VAS maximun pain scores and QuickDASH-Compulsory scores, Hierarchical Lineal Modeling (HLM) models were used. Gender and age were included as independent variables in the model. A time variable was included to determine if the evolution of the dependent variable fits a certain pattern. The spline model was used to evaluate time points where the slope changes. The fitted model used between-patient variability as a random effect. The significance level of the statistical tests was set at P < 0.05.

## Results

All 18 tennis players returned to play at an average of 3.31 ± 0.61 months post-intervention.

### Outcome at 6 months

All clinical and MRI evaluation after ASCs implantation are presented in Table 1.

**Table 1:** Outcome evaluation at 6 months after ASCs injection compared to baseline in 18 LET patients

Variables	Baseline^a^	6 months post procedure^a^	Mean difference (95%IC)	*P* value
VAS for maximum pain	6.28 ± 1.65	1.05 ± 0.43	5.23 (4.52–5.95)	< 0.001
QuickDASH-Compulsory	51.38 ± 12.02	12.33 ± 4.66	39.05 (35.16–42.94)	< 0.001
QuickDASH-Sport	56.94 ± 15.44	8.68 ± 8.86	48.26 (44.44–52.08)	< 0.001
Total MRI score	4.22 ± 0.26	2.22 ± 0.10	2 (1.48–2.51)	< 0.001

^a^Values are expressed ± standard deviation

**Fig. 1:**
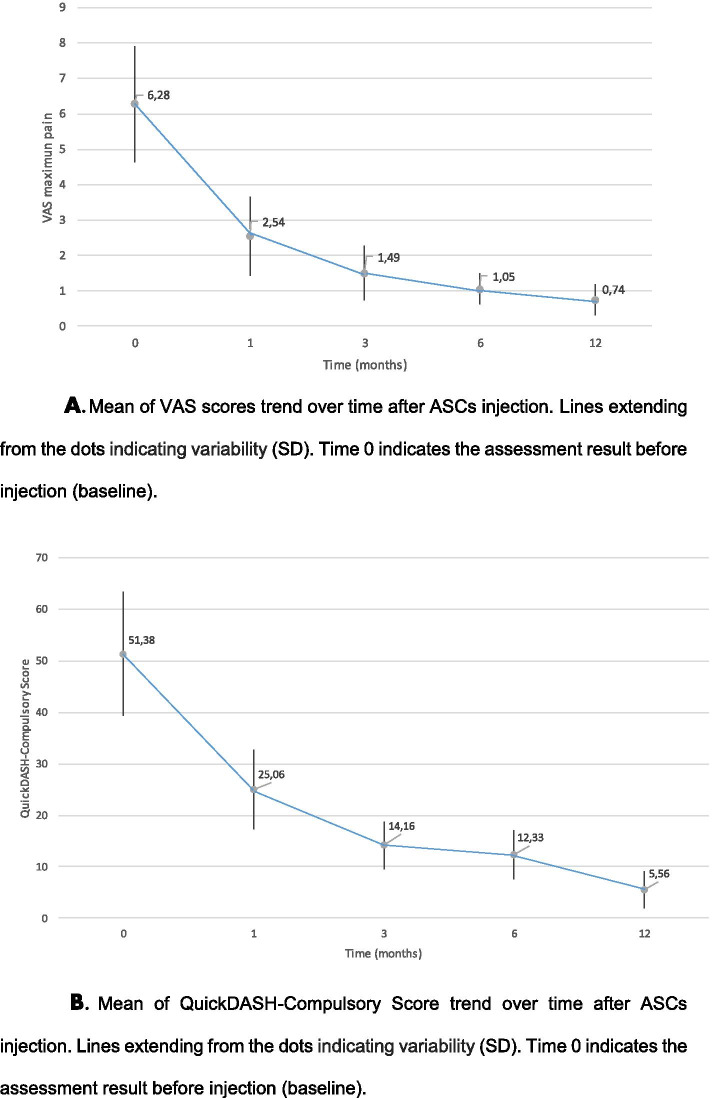
**a** Mean of VAS scores trend over time after ASCs injection. Lines extending from the dots indicating variability (SD). Time 0 indicates the assessment result before injection (baseline). **b**. Mean of QuickDASH-Compulsory Score trend over time after ASCs injection. Lines extending from the dots indicating variability (SD). Time 0 indicates the assessment result before injection (baseline)

Figure 1 a and b presented the evolution of VAS maximum pain and QuickDASH-Compulsory score over time (baseline, 1, 3, 6, and 12 months)

Table 2 shows the results of the Hierarchical Lineal Modeling (HLM) for VAS maximum pain.

**Table 2:** Hierarchical Lineal Modeling for VAS maximum pain

Model component	ß Coefficient	Standard error	*P* value
Baseline VAS (Intercept)	6.39	0.24	< 0.001
First month improvement^a^	-3.75	0.22	< 0.001
Time point for improvement trend change^a^	1.26	0.07	< 0.001
Monthly improvement after first month^a^	-0.08	0.02	0.0018
Time in months^a^	0.07	0.06	0.26
Gender	0.10	0.39	0.79
Age^b^	-0.02	0.03	0.43

^a^based on one-unit change

^b^based on one-unit change

A significant improvement in VAS after ASCs implantation (58,69% reduction) at first month was observed. After first month (modeling point of trend change = 1.26) the monthly improvement was -0.08. Gender and age were not statistically significant predictors of VAS evolution.

Table 3 shows the results of the Hierarchical Lineal Modeling (HLM) for QuickDASH-Compulsory score.

**Table 3:** Hierarchical Lineal Modeling for QuickDASH-Compulsory score

Model component	ß Coefficient	Standard error	*P* value
Baseline QuickDASH-Compulsory (Intercept)	52.70	1.70	< 0.001
First month improvement	-26.33	1.31	< 0.001
Time point for improvement trend change	1.33	0.06	< 0.001
Monthly improvement after first month	-0.98	0.14	< 0.001
Time in months	0.63	0.44	0.16
Gender	-0.21	3.05	0.95
Age	-0.13	0.20	0.52

A significant improvement in the score after ASCs implantation (50,04% reduction) at 1 month was observed. After the first month (modeling point of trend change = 1.33) the monthly improvement was -0.98. Gender and age were not statistically significant predictors of QuickDASH-Compulsory score evolution.

### Patient Satisfaction

At 3 months post-injection, 8 patients were very satisfied with their overall experience, 9 patients were satisfied, while 1 patient was neutral. At 6 months evaluation 15 patients were very satisfied with the procedure, 2 were satisfied and the same patient was neutral. At 12 months, Of the 18 players, 17 very satisfied with both the procedure and the outcome. They were ready to undergo the procedure again if needed and 17 of 18 patients would recommend this procedure.

### Adverse events

Subcutaneous haematoma in two different patients with further induration were observed in the site of tissue harvest. Both got resolved without further treatment. In one patient the induration persisted during 1 week whereas in the second 1 month. A mild discomfort at the injection site was observed in 6 patients. No other complications, residual symptoms or disability in the periumbilical zone was observed or reported. There were no infections, nor other know untoward effects.

**Fig. 2:**
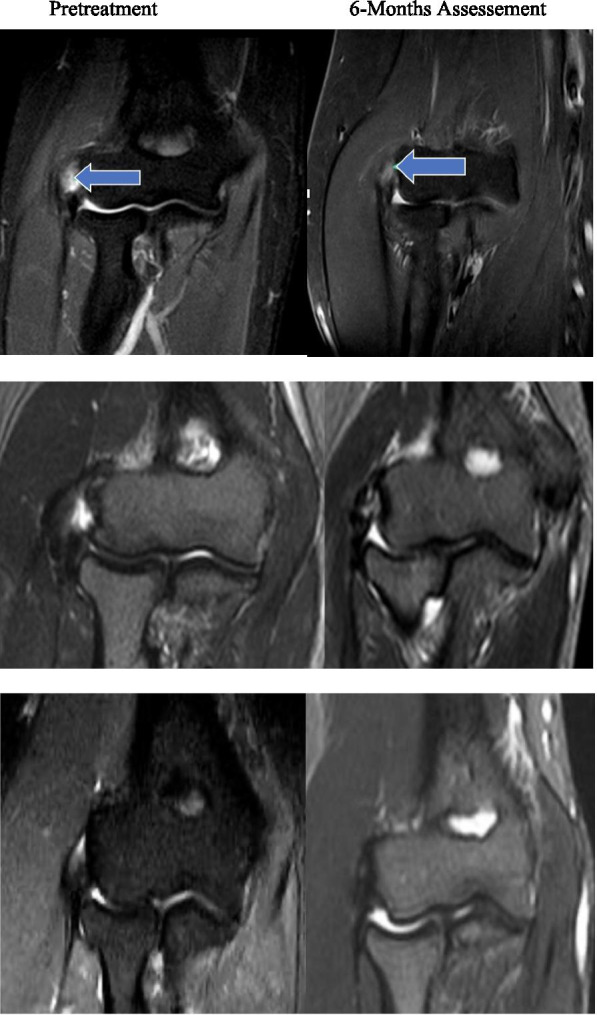
STIR Magnetic resonance imaging (MRI) results after autologous adipose derived stromal cells injection. Top row: patient 2´s score was 4 (tendinopathy [TD], 2; tear, 2) at pretreatment, and 3 (TD, 2; tear, 1) at 6 months assessement. Middle row: Patient 8´s score was 6 (TD, 3; tear, 3) at pretreatment, and 3 (TD, 2; tear, 1) at 6 months assessement. Botton row: Patient 17´s score was 3 (TD, 2; tear, 1) at pretreatment, and 2 (TD, 1; tear, 1) at 6 months assessement. Total MRI scores ranged from 2 to 6 (best to worst)

MRI study of patients: baseline and 6 months follow-up studies. Figures shows the (partial) structural improvement (Table 4, Fig. 2).

**Table 4:** Tendinosis, tear and total scores pre and 6 months post-injections

	Tendinosis		Tear		Total	
	Pre-Inj	6 Months Post-Inj	Pre-Inj	6 Months Post-Inj	Pre-Inj	6 Months Post-Inj
Mean	2.3	1.2	1.9	1.1	4.2	2.2
Std. Deviation	.69	.41	.71	.32	1.11	.41

## Discussion

The major finding of this study was a drastic improvement in pain and functional scores during the first month after ultrasound guided injections of ASCs. After this initial effect there was a decelerating trend in outcome measurements results over time. At a slower rate the functional scores continued to improve during the 12-month follow-up of the players. The structural repair of the tendon was evidenced by MRI at 6-month. This study showed that ASCs injections for recalcitrant LET, is possible, safe and of promising efficiency.

Our study is in some ways similar to others previously published literature. Wang et.al. implanted cultured autologous tenocytes under ultrasound guidance in patients with chronic resistant LET who had previously undergone unsuccessful nonsurgical treatment. Initially the study follow-up was of 12 months and in a second evaluation they published their results over a supplementary 4.5 years follow-up period the study concluded that autologous tenocyte injection improved clinical function and MRI tendinopathy scores up to 5 years [40]. Contrary to previous studies, we did not need to obtain a biopsy from the patellar tendon and were therefore did not expose our patients to the possible associated morbidities. Lee et.al. conducted a study of lateral epicondylosis by using allogeneic adipose-derived mesenchymal stem cells [24]. They reported that this therapy was safe and effective in improving elbow pain, functional performance, and structural defects by 12 months post-intervention. An advantage of this study was that they did not need to harvest cells before injecting the patients, and they did not detect any immunologic rejection response in any of the subjects. Connell et al. [8] published a treatment of lateral epicondylosis using cultured skin-derived tenocyte-like cells. Ultrasonography showed a healing response with a decrease in number of tears, number of new vessels and tendon thickness. Of the 12 patients, 11 had a satisfactory outcome, and only one patient proceeded to surgery after failure of treatment three months after injection. Singh et al. [33] using bone marrow aspirate concentrate (BMAC) and evaluating at 1,5 and 3-month post-injection, concluded that the treatment of LET in patients with a single injection of BMAC showed a significant improvement in short to medium term follow-up. Although the optimal cell number to treat this condition is not known, a potential advantage of our procedure was that we were able to implant a higher number of mesenchymal stromal cells compared to the number of stromal cells provided by BMAC procedures, that yields less than 1% of mesenchymal stromal cells per procedure [34].

The idea of stem cells as cell replacement has changed to that of paracrine provider. Three potential mechanisms for MSCs have been proposed: (i) MSCs can secrete various cytokines and growth factors to adjacent cells, named as a paracrine effect, which may elicit cellular proliferation and damaged tissue healing [7] (ii) MSCs act through immunomodulatory properties that could improve healing of injured tissues [28] and (iii) MSCs can differentiate into targeted cell types and contribute to wound repair [31]. In the present study, clinical improvement was already present at early stages after injection. The timing of this finding supports the first two mechanisms of action prior to regeneration at the cellular or structural levels. After this time period, improvement continued but at a slower rate. There was an apparent delay between the observed clinical improvement and the structural one as showed in the 6 months post-injection’ MRI. Incomplete healing was observed in most of the images. This observation supports the idea that a continued healing and remodeling process would continue after clinical recovery and MRI evaluation period. Our study agrees with Vermeulen et al. [37] work, where complete resolution of an intramuscular tendon injury on MRI is not necessary for a successful return to play. The latter authors found that 56% of the clinically recovered hamstring intramuscular tendon injuries showed a partial or complete thickness discontinuity on MRI.

Given the hypocellular nature of tendons, the application of cell-based therapies is quite intriguing and several challenges need to be addressed. Tendon cell populations have been shown to include tendon stem/progenitor cells (TSPCs). These cells fulfill the universal criteria of mesenchymal stromal cells MSCs: clonogenicity, multipotency and self-renewal [43]. Tendon healing frequently encompasses the differentiation of resident TSPCs toward a phenotype resembling that of activated fibroblasts [5]. In that regard, the use of ASCs envisions a modulation of the inflammatory environment targeting a potential promising shift from pro-inflammatory and pro-fibrotic, to pro-regenerative cellular responses, leading to a potential reduced infiltration of inflammatory cells and ordered deposition of extra cellular matrix components [10].

ASCs are widely applied in pre-clinical and clinical investigations with the therapeutic purpose of regenerative medicine and immunomodulation. Either the heterogenous whole stromal vascular fraction (SVF), which is yielded via mechanical and enzymatic digestion, or further isolated and expanded ASCs can be directly used in clinical studies. Most of the current clinical trials exclusively use the stromal vascular fraction (SVF), rather than cultured, plastic-adherent ASCs; this is probably due to restrictions on the use of cultured cells in humans [23]. Like ASCs, the SVF is harvested from adipose tissue but is administered directly after tissue digestion and lavage of liberated cells, without cell culture. Usuelli et al. [35] showed that an intra-tendinous adipose-derived stromal vascular fraction injection provides a safe, efficacious treatment for achilles tendinopathy in a controlled trial at 6-months follow-up. Garza et al. [14] used intra-articular SVF cells derived from liposuction to effectively treat knee osteoarthritis and evaluated the outcome at 12-month follow-up. Koh et al. [20] had encouraging results in treating patients undergoing high tibial osteotomy using SVF cells obtained from the infrapatellar fat pad. ASCs were reported to efficiently enhance the regeneration of cartilage and appears to be an effective therapy for knee osteoarthritis. ASCs were even reported to having the potential to prevent disease progression in a recently study published by Yokota et al. [42]. Jo et al. [17] used ASCs to treat rotator cuff disease. They performed intra-tendinous injection of autologous expanded cells in patients with partial thickness rotator cuff tears. Using MRI and second look arthroscopies reported a significant decrease in articular- and bursal-side defects. They also found improved shoulder function and pain with no adverse effects.

Little has been published in the peer-reviewed literature on the costs of cell-based therapies. As these treatments result in high costs to patients that are generally paid entirely out-of-pocket, it will be critical to establish an appropriate and sustainable pricing and reimbursement model in the future. To our knowledge, no studies have assessed the cost-effectiveness of stromal cell therapies for musculoskeletal conditions [23].

Sanders et. al. [30] suggested that those LET without symptom resolution within 6 months of onset will tend to have a more prolonged course possibly requiring surgical intervention. In our study, the mean symptomatic time in participants was 7,3 months, clearly pointing out at the chronic aspect of the condition. Although full-thickness tear of the common extensor origin were excluded, we definitively think that the included players were not affected by a self-limiting LET. Moreover, costs of stromal cells procedures, even though representing a financial load, should be compared to the high costs and potential risks of surgical intervention.

ASCs injections of the present study were well tolerated with no adverse effects on the site of injection. We have not observed joint effusion or skin hypersensitivity reactions as previously reported [24]. It should be noted that joint contamination after ultrasound guided injections may be inevitable [13]. Only a mild discomfort at the injection site was observed in 6 patients most likely related to the intra-tendinous injection per se. There were only two patients with a mild hematomas and induration in the site of the harvest of the adipose tissue. Based on the current clinical trials, cultured MSCs therapy appears to be safe [22, 27, 41].

A strength of this study was to include stratified patients according to MRI grade of tendinopathy. The use of MRI evaluation allowed us to obtain non-invasive information related to improvement of the structural integrity of the damaged tendon and to evaluate repair of the tendinopathy after ASCs implantation. A limited number of studies have attempted to use radiological measures to assess structural changes within the tendon after treatment [8, 24, 40]. As propose by Bhabra et. al., only pathoanatomical graduation of the disease gives reliability to studies but unfortunately this is only obtained by invasive tissue biopsy [4].

The present investigation has some limitations related to (i) the relatively low number of patients, (ii) the lack of a control group and (iii) outcome assessors were not blinded. Another limitation of this study is that MRI studies are not able to distinguish if the defect-filling tissues of the LET were differentiated ASCs already secreting collagen fibers or just proliferated tissue from damaged local tendon. We suggest that this would be incorporated in future studies, especially those not including a control group/condition. A longer follow-up is also required to see any eventual adverse effects like potential calcification in tendon or tumorigenesis. Indeed, one of the main concerns about safety of stromal cells use is the potential for carcinogenesis. In this context, Rubio et al. [29] in their first report demonstrated that cellular transformation could happen in human adult mesenchymal cells. They showed that after a long-term in vitro culture of MSCs, some of the cells underwent spontaneous carcinogenic transformation. However, this article was retracted by the authors after its’ publication. They manifested a probably cross-contamination artifact and the inability to reproduce their own findings [11].Klopp et al. [19] found no evidence of MSCs related tumor growth in more than 1,000 patients treated by several conditions. Therefore, it looks like carcinogenic potential of MSCs might not be as high as previously thought. However, the authors of the present study wanted to mention the retracted paper of [29] in a concern of total transparency.

## Conclusion

Tennis players with recalcitrant LET showed significant clinical improvement already at 1 month after injection of autologous ASCs and structural repair at the origin of the common tendon origin at 6 months. The clinical improvements were observed until 12 months post-injection. Future well-designed randomized trials are needed to define the role of cell therapy in treating LET.

## Data Availability

Available.

## Abbreviations

LET: Lateral elbow tendinopathy
MRI: Magnetic resonance imaging
AT: Adipose tissue
ASCs: Adipose derived mesenchymal stromal cells (expanded)
MSCs: Mesenchymal stromal cell (expanded)
TSPCs: Tendon stromal progenitor cells
SVF: Stromal vascular fraction (non-expanded)
BMAC: Bone marrow aspirate concentrate
VAS: Visual analog scale
DASH: Disabilities arm shoulder and head
HBSS: Hanks balanced salt solution
HLM: Hierarchical linear modeling
CD: Cluster differentiation
